# A tutorial on using the paired *t* test for power calculations in repeated measures ANOVA with interactions

**DOI:** 10.3758/s13428-022-01902-8

**Published:** 2022-08-24

**Authors:** Benedikt Langenberg, Markus Janczyk, Valentin Koob, Reinhold Kliegl, Axel Mayer

**Affiliations:** 1 Bielefeld University, Bielefeld, Germany; 2 University of Bremen, Bremen, Germany; 3 University of Potsdam, Potsdam, Germany

**Keywords:** Repeated measures ANOVA, Power, Effect sizes, Interactions

## Abstract

The a priori calculation of statistical power has become common practice in behavioral and social sciences to calculate the necessary sample size for detecting an expected effect size with a certain probability (i.e., power). In multi-factorial repeated measures ANOVA, these calculations can sometimes be cumbersome, especially for higher-order interactions. For designs that only involve factors with two levels each, the paired *t* test can be used for power calculations, but some pitfalls need to be avoided. In this tutorial, we provide practical advice on how to express main and interaction effects in repeated measures ANOVA as single difference variables. In particular, we demonstrate how to calculate the effect size Cohen’s *d* of this difference variable either based on means, variances, and covariances of conditions or by transforming ${\eta _{p}^{2}}$ or ${\omega _{p}^{2}}$ from the ANOVA framework into *d*. With the effect size correctly specified, we then show how to use the *t* test for sample size considerations by means of an empirical example. The relevant R code is provided in an online repository for all example calculations covered in this article.

A priori power calculations play a crucial role in psychological studies, as they allow researchers to determine the required sample size to detect an effect of a particular size with a desired probability, under the assumption that this effect actually exists (e.g., Cohen, 1988). As such, the utility of power analyses has long been known and advocated (e.g., Wilkinson & Task Force on Statistical Inference, American Psychological Association, Science Directorate, 1999). Yet, the seminal work by Cohen (1962) already revealed that most studies in psychology lack the adequate power to detect an effect of interest, and this state does not seem to have changed much (Maxwell, 2004; Sedlmeier & Gigerenzer, 1989; Vankov, Bowers, & Munafò, 2014).

Issues of power analyses have received even more attention in the realm of the often-discussed “replication crisis”, that is, the observation that many published results cannot be replicated (e.g., Open Science Collaboration, 2015). Among other problems with underpowered studies (summarized in, e.g., Brysbaert, 2019; Fraley & Vazire, 2014), the probability of replicating a result increases with the power of the original study (Ioannidis, 2005). Some funding agencies and journals explicitly require authors to include power considerations in their submissions, related questions are posed by reviewers, and it was even suggested to base quality judgments of journals (among other criteria) on the (mean) power of the studies published within them (Fraley & Vazire, 2014).

Yet, power analyses come with some obstacles once going beyond situations where two groups or conditions can be compared via *t* tests, and this seems in particular to be true for within-subject designs, where participants provide data for more than one condition (and often on multiple trials per condition, as is typical for experiments in cognitive psychology).^1^ Of course, there exists a wide range of articles and books on power analysis (e.g., Cohen, 1988; Keselman et al., 1998; Maxwell, Kelley, & Rausch, 2008; Olejnik & Algina, 2000; Perugini, Gallucci, & Costantini, 2018; Steiger, 2004), as well as software packages like G*Power (Faul, Erdfelder, Lang, & Buchner, 2007), *Superpower* (Lakens & Caldwell, 2021), or *pwr* (Champely et al., 2020). Still, the correct specification of arguments is often unclear and this leaves researchers unsure whether their calculations are actually valid or not.

In this tutorial, we focus on the power analysis for a certain use case that often occurs in experimental cognitive psychology: Calculating the power for interactions or main effects in a repeated-measures analysis of variance (RM-ANOVA), where each involved factor has only two levels. Despite the narrow focus, such designs are very common, for instance, in the field of cognitive control from where we also draw the example introduced below. Although our main motivation for writing this tutorial aims at power calculation for interactions, power calculation for main effects follows a highly related logic. This tutorial thus provides practical advice, software code, and formula to perform the necessary calculations for both main and interaction effects.

## The focus of this tutorial

According to our experience in methodological consulting, there exists uncertainty about whether power calculations (for interactions) in within-subject designs can be performed with standard software packages such as G*Power (Faul et al., 2007) or only achieved via simulations. For the special case of only two levels on each factor (i.e., a $2 \times 2 \times 2 \dots$ design), the main and interaction effects can be conceived of as differences or “differences of differences”. These effects thus boil down to a simple difference variable for which the power analysis can then be done in the framework of a paired-samples *t* test. The advantage of this approach is clear: Power calculations for the *t* test are relatively straightforward and solely require the specification of an expected effect size in terms of Cohen’s *d*, instead of ${\eta _{p}^{2}}$ that is often used in the context of RM-ANOVA. Although it has been described in numerous standard texts how main and interaction effects can be expressed as differences (of differences; e.g., Aiken & West, 1991; Cohen, Cohen, West, & Aiken, 2013; Judd, McClelland, & Ryan, 2017; Maxwell & Delaney, 2004), we find that researchers rarely make use of this fact and the paired-samples *t* test for power calculations. This tutorial provides step-by-step instructions on how to express main and interaction effects as a difference (of differences) variable and how the effect size of such variables can be used to perform a power analysis.

Researchers can generally select one of three strategies to derive effect sizes. First, they can formulate a specific expectation about the means and (co)variances of the dependent variables in a (multi-)factorial experimental design. Such an expectation can be informed by expertise or by a re-analysis of (multiple) data sets, for instance, in case one considers replicating an experiment or extending previous observations in a follow-up study. Second, they can have knowledge about previously reported effect sizes, for instance, because of an available meta-analysis or a review of the relevant literature. Third, they can rely on conventions prevalent in a certain field of research. Most prominently for psychology are the suggestions of Cohen (1988). In particular, Cohen proposed that the effect-size measures *d* = 0.2, *d* = 0.5, and *d* = 0.8 can be considered as small, medium, and large, respectively, and identical labels were assigned to the effect-size measures ${\eta ^{2}_{p}}=.01$, ${\eta ^{2}_{p}}=.06$, and ${\eta ^{2}_{p}}=.14$ in the context of ANOVAs. It is important to note – and this will be elaborated on below – that it is *not* correct (and not even close to correct) to just use the semantic labels and conduct a power analysis for a *t* test with a large effect size *d* = 0.8 to compute the power for a large interaction effect with ${\eta _{p}^{2}} = .14$. In addition, although Cohen’s conventions are frequently used, they were meant to be a “last resort” strategy in case there is limited knowledge about the expected data pattern or effect size (see Correll, Mellinger, McClelland, & Judd, 2020, for a recent elaboration on this issue). Interestingly, meta-analyses have shown that typical effect sizes in psychological research are around *d* = 0.4, thus smaller as a medium effect according to Cohen’s labels (Camerer et al., 2018; Open Science Collaboration, 2015; see also Brysbaert, 2019). Furthermore, a study that replicated numerous original studies has reported even smaller effect sizes in the order of *d* = 0.15 (Klein et al., 2018). These findings align with the work by Schäfer and Schwarz (2019), who also found that effects from replications of studies were considerably smaller as compared to the original studies (see Janczyk et al., 2022, as an example). Thus, we urge researchers to make an educated decision regarding the expected effect size when performing power analyses.

Strategies 1 and 2 are based on estimates from previous studies, which then serve to formulate effects at the population level. For Strategy 3, an effect size is directly specified at the population level. Importantly, Strategies 2 and 3 require converting ${\eta _{p}^{2}}$/${\widehat {\eta} _{p}^{2}}$ into the effect measure *d*/$\widehat d$ for a power analysis in the *t* test framework.^2^ Of course, the various effect size measures can be converted into each other (as also implied by the conventions of Cohen), but this is (a) not trivial and (b) bears the potential of using the wrong transformation formula (i.e., to confuse the transformation for within- and between-subject designs). Below we demonstrate how to determine *d* for the three strategies and point out common pitfalls. We will also include the effect size measure ${\widehat \omega _{p}^{2}}$, which is less common, but often recommended due to its smaller bias (e.g., Carroll & Nordholm, 1975; Keselman, 1975).^3^

The tutorial is structured as follows: (1) In the upcoming section, we introduce an example for a 2 × 2 × 2 within-subject design that we use throughout this article to demonstrate calculations. We do not provide an extensive review on power analyses for every possible design, but focus on interactions and main effects in within-subject designs with two-level factors. (2) Afterward, we provide a step-by-step tutorial on how to express main and interaction effects in different designs as a single difference variable based on the dependent variables in a multi-factorial, repeated measures experimental design (Strategy 1). We show how to calculate the mean, variance, and effect size Cohen’s *d* for the difference variable using the means, variances, and covariances of the dependent variables. We explain how the effect size of the difference variable can then be used to perform a power analysis for the main and interaction effects. (3) In the section thereafter, we describe how to correctly convert ${\eta ^{2}_{p}}$/$\hat {\eta }_{p}^{2}$ (or ${\widehat {\omega} _{p}^{2}}$) to Cohen’s *d*/$\hat d$ in order to use the *t* test for power analyses (Strategies 2 and 3). We further describe challenges and pitfalls in the calculation and highlight that general rules of thumbs should be avoided (e.g., a medium ${\eta _{p}^{2}} = .06$ does not correspond to a medium Cohen’s *d* = 0.50 in case of within-subject designs). Along with the present tutorial, we provide software code in an online repository (https://osf.io/87j5m/) and the R package *powerANOVA* (Langenberg, 2022) that comes with a graphical user interface and implements the calculations covered in this tutorial. The user interface is easy to use and will not be explained in this tutorial. Installation instructions can be found on the corresponding GitHub page (https://github.com/langenberg/powerANOVA).

## A motivating example

Conflict tasks are often used in cognitive psychology to investigate how human performance is affected by task-irrelevant stimuli or stimulus features. For example, in the Eriksen flanker task (Eriksen & Eriksen, 1974), participants respond to a centrally presented target stimulus (e.g., the identity of a letter S vs. H) with a left or right key press. The critical manipulation is that the target is surrounded by other letters, the flankers, that either signal the same response on *congruent* trials (e.g., SSSSS) or the other response on *incongruent* trials (e.g., HHSHH). Response times (RTs) are typically longer (and often error rates are higher) in the incongruent compared to congruent condition – the congruency effect (CE). Another example is the Simon task (Simon & Rudell, 1967; for a review, see, Hommel, 2011). Participants have to respond, for example, to the identity of a letter, but this target stimulus is presented at a left or right location. If the (task-irrelevant) location is the same as the required response, this would be a *congruent* trial (e.g., the letter H requires a left response and the stimulus is presented on the left side). However, if the (task-irrelevant) location is different than the required response, this would be an *incongruent* trial (e.g., the letter H requires a left response and the stimulus is presented on the right side). Here, a CE is observed as well.

**Fig. 1:**
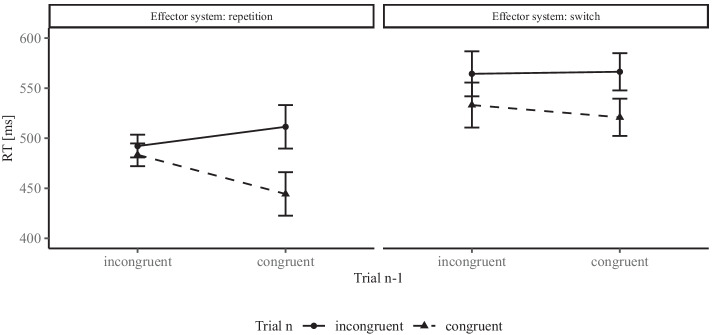
Illustration of the motivating example: a 2 × 2 × 2-interaction reflecting the difference in the congruency sequence effect (CSE). Mean response times (RT) in milliseconds (ms) are depicted as a function of congruency in trial *n* − 1, congruency in trial *n*, and effector system repetition (vs. switch). When the effector system was repeated, mean RTs were 492 (*SD* = 82) and 483 ms (*SD* = 85, incongruent vs. congruent) when the previous trial was incongruent and 511 (*SD* = 75) and 444 ms (*SD* = 88) when the previous trial was congruent. When the effector system was switched, mean RTs were 564 (*SD* = 107) and 533 ms (*SD* = 101) when the previous trial was incongruent and 566 (*SD* = 102) and 521 ms (*SD* = 107) when the previous trial was congruent. *Error bars* are confidence intervals based on the *t* tests comparing the congruent and incongruent conditions of trial *n*

The size of the CE depends on several factors. One of the most often investigated factors is recent trial history, starting with work by Gratton, Coles, and Donchin (1992). In this line of research, the congruency of the preceding trial *n* − 1 is considered in addition to the congruency of the current trial *n*. The typical observation is that the CE is larger if trial *n* − 1 was congruent, compared to when it was incongruent (see the left panel of Fig. 1 for an illustration). This observation is known as the congruency sequence effect (CSE) and has been replicated many times (e.g., Praamstra, Kleine, & Schnitzler, 1999; Schmidt & Weissman, 2014; Stürmer, Leuthold, Soetens, Schröter, & Sommer, 2002; Wühr, 2004; for a review, see, Egner, 2007). The standard analysis approach would now be a 2 × 2 RM-ANOVA with trial *n* congruency and trial *n* − 1 congruency as repeated measures, with a particular interest on the two-way interaction.

In principle, another independent variable could of course be added. Janczyk and Leuthold (2018), for example, signaled *N* = 36 participants on each trial whether they were to respond manually or with their feet. Of interest was whether the effector system repeated or switched from trial *n* − 1 to trial *n* (see Fig. 1). In this case, a 2 × 2 × 2 RM-ANOVA with trial *n* congruency, trial *n* − 1 congruency, and effector system repetition (vs. switch) as repeated measures would be required.

These examples are typical experiments from cognitive psychology. The design often includes two (or three or even more) repeated measures factors with two levels each. In the following sections, we use data from Experiment 2 (using a Simon task) of Janczyk and Leuthold (2018). This experiment followed a 2 × 2 × 2 design with the three factors congruency in trial *n* (factor A: A1 = incongruent, A2 = congruent), congruency in trial *n* − 1 (factor B: B1 = incongruent, B2 = congruent), and effector system (factor C: C1 = repetition, C2 = switch), but we show how the calculations generalize to even more complex designs (i.e., $2\times 2 \times 2 \times \dots$). The means of each of the cells and the covariances can be found in Table 1.

**Table 1:** Means, variances, and covariances for the motivating example, that is, Experiment 2 of Janczyk and Leuthold (2018)

Means				Covariances								
				Trial *n*	incong.	incong.	incong.	incong.	cong.	cong.	cong.	cong.
				Trial *n* − 1	incong.	incong.	cong.	cong.	incong.	incong.	cong.	cong.
Trial *n*	Trial *n* − 1	Effector	RT	Effector	rep.	switch	rep.	switch	rep.	switch	rep.	switch
incong.	incong.	rep.	492		6726							
incong.	incong.	switch	564		6855	11387						
incong.	cong.	rep.	511		5237	6047	5608					
incong.	cong.	switch	566		6136	9270	5878	10314				
cong.	incong.	rep.	483		6400	6971	5190	6176	7202			
cong.	incong.	switch	533		6018	8551	4909	7833	6583	10125		
cong.	cong.	rep.	444		5976	5992	4599	5846	6321	6033	7708	
cong.	cong.	switch	521		6815	10072	5925	9357	7361	9312	7058	11413

cong. = congruent; incong. = incongruent; rep. = repetition

## Strategy 1: Means and covariances approach

In this section, we show how to express main and interaction effects in a multi-factorial, repeated-measures design as difference variables and how power calculations relate to the mean and the variance of these difference variables. Using the mean and variance, the effect size of the difference variable can be expressed in terms of Cohen’s *d*, which can, in turn, be used for sample size and power calculations. With the following subsections, the complexity of the considered effects gradually increases, starting with a simple comparison of two means and ending with the interaction effects in a 2 × 2 × 2 design. Each subsection consists of two steps: (1) The main and interaction effects are expressed as differences between conditions and the mean and variance of this difference variable is calculated. (2) Based on these values, the effect size measure Cohen’s *d* is calculated and plugged into a procedure for performing the sample size and power analysis using R. We provide detailed R code for all of the examples covered in this tutorial in the accompanying online repository. For a more comprehensive introduction on RM-ANOVA and contrast coding, we would like to refer the reader to standard texts, such as Aiken and West (1991), Cohen et al. (2013), Judd et al. (2017), and Maxwell and Delaney (2004).

In the following subsections, we use the data from Experiment 2 of Janczyk and Leuthold (2018). The left part of Table 1 provides the means of each experimental condition. The right part of the table provides the variances and covariances of the original data. The values are organized as a matrix (thus a covariance matrix) of the pairwise covariances between the dependent variables. For instance, the covariance between the RT when trial *n* was congruent, trial *n* − 1 was congruent and the effector was repeated and RT when trial *n* was congruent, trial *n* − 1 was congruent and the effector switched was 7058 (last row, second last column). Covariances between experimental conditions are important, because they affect the power of hypothesis tests (as will be shown below). In fact, correlations between dependent variables is the key difference between repeated measures ANOVA and between-subject ANOVA (e.g., Liesefeld & Janczyk, 2022). The  function in R provides one way of calculating the covariance matrix of a data set. The file  in the online repository provides example code to calculate this matrix for the original (already pre-processed) data set. In what follows, we use the means, variances, and covariances of the study by Janczyk and Leuthold (2018), with the simplifying assumptions of (1) an equal variance for all conditions *σ*^2^ = 9000 (i.e., approximately the mean variance across the variables in the study, see Table 1) and (2) a covariance between the variables of *σ*_cov_ = 7200 and thus a correlation of *ρ* = 0.8 (i.e., approximately the mean correlation and covariance from the original data). The assumption of equal variances and covariances is also referred to as compound symmetry. The provided R code in this article and the online repository will also use equal variances and covariances. However, the code is very generic and can easily be altered to allow any arbitrary covariance matrix.

We want to highlight that the means and covariances (and thus effect sizes) calculated in this section are based on sample estimates from the study by Janczyk and Leuthold (2018). For power calculations, we have to assume that we know the population parameters and we here do so to illustrate the calculations. In practice, we should not rely on an estimate from a single study, but we should rather collect multiple estimates (e.g., from the literature) to obtain a clearer picture. Sometimes, however, there may not be more information available. In this case, we need to use the few information available. Additionally, one should be aware that Cohen’s *d* overestimates the true effect size. By equating estimators with the true population parameters, we might thus slightly underestimate the required sample size to achieve a desired level of power. Bias corrections have been developed by, for instance, Hedges (1981) and can be used as an alternative (see also Goulet-Pelletier & Cousineau, 2018).

Lastly, we exclusively focus on a significance level of *α* = .05, as this convention is most often used in the field of psychology. We would like, however, to point out that this convention has been criticized and researchers have advocated lower significance levels, such as *α* = .005 (Benjamin et al., 2017; Miller & Ulrich, 2019). Lakens et al. (2018) further proposed not to use a default significance level at all and that researchers should make a sound decision based on the individual study. A similar argument applies to the specification of the desired power, which we set to 1−β = .8 throughout this article. We encourage researchers to think about and justify their choices of the significance level and the power in their studies.

### Comparing two means

#### Step 1: Calculating the mean and the variance

We will start with a very simple example on how to calculate the required sample size when comparing two means and how this can be expressed in terms of the mean and the variance of the difference between both conditions. Imagine we want to perform a simple test to determine whether RTs in the motivating example differ when trial *n* was incongruent, trial *n* − 1 was incongruent as well, and the effector system was repeated (A1B1C1) versus when trial *n* was congruent, trial *n* − 1 trial was incongruent, and the effector system was repeated (A2B1C1). The difference variable can then be defined as

1 $$\begin{array}{@{}rcl@{}} X = Y_{\text{A1B1C1}} - Y_{\text{A2B1C1}} \end{array}$$

where *X* is the RT difference, and *Y*_A1B1C1_ and *Y*_A2B1C1_ are the RTs in the corresponding conditions. The mean of *Y*_A1B1C1_ is *μ*_A1B1C1_ = 492 and the mean of *Y*_A2B1C1_ is *μ*_A2B1C1_ = 483 (see Table 1). The variance in both conditions is *σ*^2^ = 9000 and the covariance is *σ*_cov_ = 7200. The mean and variance of the difference variable *X* are then:

2 $$\begin{aligned}\mu_X&=\mu_{A1B1C1}-\mu_{A2B1C1}\\&=492-483=9\end{aligned}$$

3 $$\begin{array}{@{}rcl@{}} \\ {\sigma_{X}^{2}} &= &\sigma^{2} + \sigma^{2} - 2\cdot \sigma_{\text{cov}} \\ &=& 9000 + 9000 - 2\cdot 7200 = 3600 \end{array}$$

#### Step 2: Performing the power analysis

It is now possible to calculate the effect-size measure Cohen’s *d* from the mean and the variance, which can then be used for sample size and power calculations:

4 $$\begin{array}{@{}rcl@{}} d_{X} &=& \frac{\mu_{X}}{\sigma_{X}} \\ &=& \frac{9}{\sqrt{3600}} = 0.15 \end{array}$$

The size of the effect is thus even less than small, following the conventions of Cohen (1988).

This effect size is calculated from a sample and probably does not match the effect size in the population. For the following analysis, however, we assume that this is the population effect size. We can then use this effect size and the *t* test to calculate the required sample size to achieve a power of 1−β = .8. In particular, we use a two-sided *t* test with *α* = .05 and obtain that we would need a sample size of at least *N* = 351 participants to detect an effect of *d*_*X*_ = 0.15 if this was indeed a true (but rather small) effect.

Many software packages offer the possibility to calculate the required sample size for a *t* test and so does R (R Core Team, 2021) with the function  . The following command can be used to perform the above calculations: 
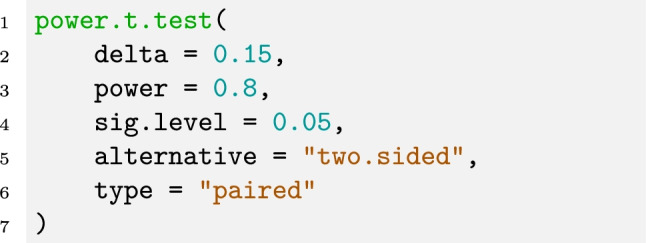


The argument  takes the effect size *d*,  is the desired power level,  is the desired significance level,  indicates if the *t* test is one- or two-sided, and  is the type of the *t* test.

As a side note, the function also provides an argument  , which can be used if  is the mean of the difference variable before dividing by the standard deviation. Hence, the above command is equivalent to the following command: 
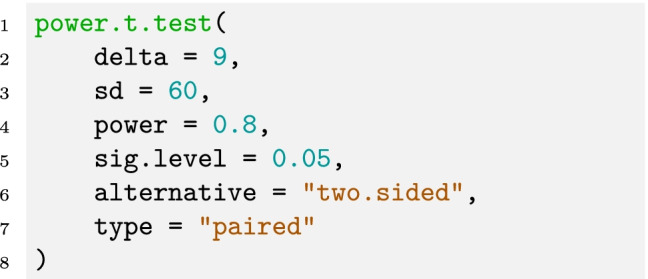


### 2 × 2 design: Main effects

#### Step 1: Calculating the mean and the variance

In the previous example, we used RTs from only two conditions. For the next example, we increase the complexity of the difference and consider a subset of the factors, that is, we use the conditions where the effector system was repeated. This leaves us with a 2 × 2 design consisting of the factors A and B. Although not as obvious as in the previous example, we can still use the *t* test for power analysis in this case.

Assume we want to investigate the main effect of trial *n* (factor A). The main effect of factor A can be expressed as the sum of all conditions where trial *n* trial was incongruent (A1) minus the sum of all conditions where trial *n* was congruent (A2). Thus, the difference variable can be defined as

5 $$\begin{array}{@{}rcl@{}} X_{\text{A}} &= &(Y_{\text{A1B1}} + Y_{\text{A1B2}}) - (Y_{\text{A2B1}} + Y_{\text{A2B2}}) \\ &=& Y_{\text{A1B1}} + Y_{\text{A1B2}} - Y_{\text{A2B1}} - Y_{\text{A2B2}} \end{array}$$

and the value of the difference variable in the example is then:

6 $$\begin{array}{@{}rcl@{}} \mu_{X_{\text{A}}} &=& \mu_{\text{A1B1}} + \mu_{\text{A1B2}} - \mu_{\text{A2B1}} - \mu_{\text{A2B2}} \end{array}$$

7 $$\begin{array}{@{}rcl@{}} &=& 492 + 511 - 483 - 444 = 76 \end{array}$$

Calculating the variance is slightly more difficult. Yet, the assumption that the variances and covariances are equal across conditions simplifies the formula. With *k* denoting the number of factors in the design (i.e., *k* = 2 in the present case), the variance of the difference variable can be calculated as:

8 $$\begin{array}{@{}rcl@{}} \sigma_{X_{\text{A}}}^{2} &=& 2^{k} \cdot \sigma^{2} - 2^{k} \cdot \sigma_{\text{cov}} \\ &=& 2^{2} \cdot 9000 - 2^{2} \cdot 7200 = 7200 \end{array}$$

The formula for the variance becomes more complicated when we want to use different variances for and covariances between conditions. We provide R scripts in the online repository that can be used as a template and the user only has to insert the correct values in the covariance matrix. The following chunk of R code shows how the mean, the covariance matrix, and a contrast vector are specified in order to calculate the mean and the variance of the difference variable as defined above: 
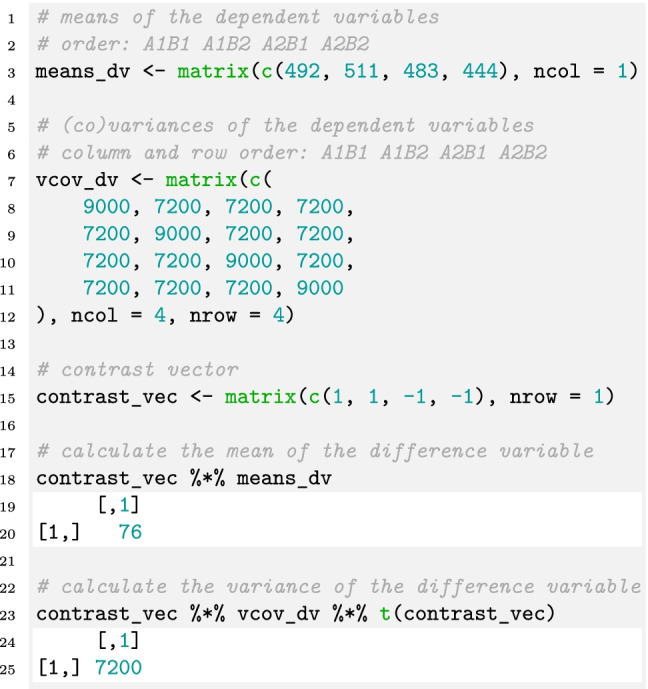


In Line 3, the means of the dependent variables are defined, which are equivalent to the means from Eq. 7. In Line 7, the covariance matrix of the dependent variables is defined, which conforms to the compound symmetry assumption. We can easily change the variances and covariances to any other value if we do not want to assume compound symmetry (with the constraint that the covariance matrix must be positive definite). Line 15 defines the contrast vector based on Eq. 6. The first two elements are 1, because the means *μ*_A1B1_ and *μ*_A1B2_ enter with a positive sign into that equation. The third and fourth elements are -1, because the means *μ*_A2B1_ and *μ*_A2B2_ enter with a negative sign. In Line 18, the mean of the difference variable is calculated by multiplying the contrast vector with the mean vector (i.e., the cross-product) and the result is printed in Line 20. In Line 23, the variance of the difference variable is calculated by pre- and post-multiplying the covariance matrix with the contrast vector and the result is printed in Line 25.

#### Step 2: Performing the power analysis

We again use the mean and variance of the difference variable to calculate Cohen’s *d*:

9 $$\begin{array}{@{}rcl@{}} d_{X_{\text{A}}} &=& \frac{\mu_{X_{\text{A}}}}{\sigma_{X_{\text{A}}}} \\ &=& \frac{76}{\sqrt{7200}} \approx 0.9 \end{array}$$

Assuming that the effect size is the population effect size, we can calculate the sample size required to achieve a power of 1−β = .8. We find that we would need a sample size of at least *N* = 12 to detect the effect with a probability of 1−β = .8. The R command for this analysis is very similar as for the previous example. Only the argument  must be replaced by $d_{X_{\text {A}}} = 0.9$ (in case  is set to its default value of 1).

### 2 × 2 design: Interaction effect

#### Step 1: Calculating the mean and the variance

The interaction effect in a 2 × 2 design can be expressed in terms of a difference variable as well. In this case, the difference is, in fact, a difference of differences. It is the RT difference of the differences where trial *n* − 1 was incongruent (B1) and where trial *n* − 1 was congruent (B2) between the two levels of trial *n* (A1 minus A2):

10 $$\begin{array}{@{}rcl@{}} X_{\text{A:B}} &=& X_{\text{B | A1}} - X_{\text{B | A2}}\\ &=& (Y_{\text{A1B1}} - Y_{\text{A1B2}}) - (Y_{\text{A2B1}} - Y_{\text{A2B2}}) \\ &= &Y_{\text{A1B1}} - Y_{\text{A1B2}} - Y_{\text{A2B1}} + Y_{\text{A2B2}} \end{array}$$

For clarification, *X*_B | A1_ indicates the RT difference between the condition where trial *n* − 1 trial was incongruent and the condition where trial *n* − 1 was congruent while trial *n* was incongruent. The mean of the difference variable for our example is:

11 $$\begin{array}{@{}rcl@{}} \mu_{X_{\text{A:B}}} &=& \mu_{\text{A1B1}} - \mu_{\text{A1B2}} - \mu_{\text{A2B1}} + \mu_{\text{A2B2}} \\ &=& 492 - 511 - 483 + 444 = -58 \end{array}$$

The variance is again a bit more difficult. However, under compound symmetry, it turns out that the same formula as for the main effect can be used here (without compound symmetry, one would have to specify the exact covariance matrix, e.g., by adapting the R code of the supplemental material in the online repository):

12 $$\begin{array}{@{}rcl@{}} \sigma_{X_{\text{A:B}}}^{2} &=& 2^{k} \cdot \sigma^{2} - 2^{k} \cdot \sigma_{\text{cov}} \\ &=& 2^{2} \cdot 9000 - 2^{2} \cdot 7200 = 7200 \end{array}$$

#### Step 2: Performing the power analysis

The effect size Cohen’s *d* is then calculated as

13 $$\begin{array}{@{}rcl@{}} d_{X_{\text{A:B}}} &=& \frac{\mu_{X_{\text{A:B}}}}{\sigma_{X_{\text{A:B}}}} \\ &=& \frac{-58}{\sqrt{7200}} \approx -0.68 \end{array}$$

and the result can be considered a medium to large effect according to Cohen (1988).

Assuming that this value is the population effect size, the power calculation yields a required sample size of *N* = 19 to observe an effect of this magnitude with a power of 1−β = .8.

### 2 × 2 × 2 design: Main effects

#### Step 1: Calculating the mean and the variance

We now use the full 2 × 2 × 2 design of the example. Again, a main effect in this design with three factors with two levels each can be expressed as a single difference variable.

Assume we want to investigate the main effect of trial *n* (factor A) as before, but we use the full design now. The procedure is just as for a 2 × 2 design. We compare the sum of all conditions where trial *n* is incongruent (A1) against the sum of all conditions where trial *n* is congruent (A2). We thus define the difference variable as:

14 $$\begin{array}{@{}rcl@{}} X_{\text{A}} &= {} & (Y_{\text{A1B1C1}} + Y_{\text{A1B1C2}} + Y_{\text{A1B2C1}} + Y_{\text{A1B2C2}}) - \\ & &(Y_{\text{A2B1C1}} + Y_{\text{A2B1C2}} + Y_{\text{A2B2C1}} + Y_{\text{A2B2C2}}) \\ &= {} & Y_{\text{A1B1C1}} +Y_{\text{A1B1C2}} + Y_{\text{A1B2C1}} + Y_{\text{A1B2C2}} - \\ & & Y_{\text{A2B1C1}} - Y_{\text{A2B1C2}} - Y_{\text{A2B2C1}} - Y_{\text{A2B2C2}} \end{array}$$

The mean of the difference variable for our example can then be calculated as

15 $$\begin{array}{@{}rcl@{}} \mu_{X_{\text{A}}} &= {} &\mu_{\text{A1B1C1}} + \mu_{\text{A1B1C2}} + \mu_{\text{A1B2C1}} + \mu_{\text{A1B2C2}} - \\ & & \mu_{\text{A2B1C1}} - \mu_{\text{A2B1C2}} - \mu_{\text{A2B2C1}} - \mu_{\text{A2B2C2}} \\ &= {} & 492 + 564 + 511 + 566 - 483 - 533 - 444 - 521 = 152 \end{array}$$

and the variance can be calculated following Eq. 8 with *k* = 3:

16 $$\begin{array}{@{}rcl@{}} \sigma_{X_{\text{A}}}^{2} &=& 2^{k} \cdot \sigma^{2} - 2^{k} \cdot \sigma_{\text{cov}} \\ &=& 2^{3} \cdot 9000 - 2^{3} \cdot 7200 = 14400 \end{array}$$

In fact, the formula can be used for any number of factors as long as (1) the variances and covariances are equal and (2) the factors have only two levels each.

#### Step 2: Performing the power analysis

Using the mean and variance of the difference variable, we can calculate Cohen’s *d* as

17 $$\begin{array}{@{}rcl@{}} d_{X_{\text{A}}} &=& \frac{\mu_{X_{\text{A}}}}{\sigma_{X_{\text{A}}}} \\ &=& \frac{152}{\sqrt{14400}} \approx 1.27 \end{array}$$

which is even larger than large following the conventions of Cohen (1988).

Using the effect size as the population effect size, we calculate that we would need a sample size of *N* = 8 to achieve a power of 1−β = .8. This number is very low, due to the large effect size.

### 2 × 2 × 2 design: Two-way interactions

#### Step 1: Calculating the mean and the variance

The required calculations for the two-way interaction of, for example, factor A and B in the 2 × 2 × 2 design are very much the same as for the simpler 2 × 2 design. Only the number of involved variables is larger. The interaction effect is the difference of the differences between the incongruent (B1) and the congruent (B2) condition in trial *n* − 1 (factor B) between the incongruent (A1) and the congruent (A2) condition in trial *n* (factor A) while summing across factor C. Expressed formally, this yields

18 $$\begin{array}{@{}rcl@{}} X_{\text{A:B}} &= & X_{\text{B | A1}} - X_{\text{B | A2}}\\ &= & [(Y_{\text{A1B1C1}} + Y_{\text{A1B1C2}}) - (Y_{\text{A1B2C1}} + Y_{\text{A1B2C2}})] - \\ & &[(Y_{\text{A2B1C1}} + Y_{\text{A2B1C2}}) - (Y_{\text{A2B2C1}} + Y_{\text{A2B2C2}})] \\ &= & Y_{\text{A1B1C1}} + Y_{\text{A1B1C2}} - Y_{\text{A1B2C1}} - Y_{\text{A1B2C2}} - \\ & &Y_{\text{A2B1C1}} - Y_{\text{A2B1C2}} + Y_{\text{A2B2C1}} + Y_{\text{A2B2C2}} \end{array}$$

and the expected value of the difference of differences variable for the example can be calculated as:

19 $$\begin{array}{@{}rcl@{}} \mu_{X_{\text{A:B}}} &= & \mu_{\text{A1B1C1}} + \mu_{\text{A1B1C2}} - \mu_{\text{A1B2C1}} - \mu_{\text{A1B2C2}} - \\ & &\mu_{\text{A2B1C1}} - \mu_{\text{A2B1C2}} + \mu_{\text{A2B2C1}} + \mu_{\text{A2B2C2}} \\ &= & 492 + 564 - 511 - 566 - 483 - 533 + 444 + 521 = -72 \end{array}$$

For calculating the variance, we use the same formula as before. It does not matter whether we are dealing with a main effect or an interaction effect – the formula is the same under compound symmetry. Only the number of involved factors matters:

20 $$\begin{array}{@{}rcl@{}} \sigma_{X_{\text{A:B}}}^{2} &=& 2^{k} \cdot \sigma^{2} - 2^{k} \cdot \sigma_{\text{cov}} \\ &=& 2^{3} \cdot 9000 - 2^{3} \cdot 7200 = 14400 \end{array}$$

#### Step 2: Performing the power analysis

With the mean and the variance, Cohen’s *d* is calculated as before:

21 $$\begin{array}{@{}rcl@{}} d_{X_{\text{A:B}}} &=& \frac{\mu_{X_{\text{A:B}}}}{\sigma_{X_{\text{A:B}}}} \\ &=& \frac{-72}{\sqrt{14400}} = -0.6 \end{array}$$

Assuming this is the true effect size, we would need a sample size of *N* = 24 to achieve a power of 1−β = .8.

### 2 × 2 × 2 design: Three-way interactions

#### Step 1: Calculating the mean and the variance

Finally, we consider how to express a three-way interaction in terms of a difference. In fact, this interaction is a difference of differences of differences. In other words, the three-way interaction expresses whether the interaction *B* × *C* is different for the two levels of factor A. This difference variable can be written as:

22 $$\begin{array}{@{}rcl@{}} X_{\text{A:B:C}} &= & X_{\text{B:C | A1}} - X_{\text{B:C | A2}} \\ &= & (X_{\text{C | A1B1}} - X_{\text{C | A1B2}}) - (X_{\text{C | A2B1}} - X_{\text{C | A2B2}}) \\ &= & [(Y_{\text{A1B1C1}} - Y_{\text{A1B1C2}}) - (Y_{\text{A1B2C1}} - Y_{\text{A1B2C2}})] - \\ && [(Y_{\text{A2B1C1}} - Y_{\text{A2B1C2}}) - (Y_{\text{A2B2C1}} - Y_{\text{A2B2C2}})] \\ &= & Y_{\text{A1B1C1}} - Y_{\text{A1B1C2}} - Y_{\text{A1B2C1}} + Y_{\text{A1B2C2}} - \\ & &Y_{\text{A2B1C1}} + Y_{\text{A2B1C2}} + Y_{\text{A2B2C1}} - Y_{\text{A2B2C2}} \end{array}$$

We see that we calculate the difference between C1 and C2 in the innermost parentheses. We then calculate the difference of this difference between the two levels of B1 and B2. Finally, we calculate the differences of this difference between the two levels A1 and A2. The value of this difference for our example can be calculated as

23 $$\begin{array}{@{}rcl@{}} \mu_{X_{\text{A:B:C}}} &= & \mu_{\text{A1B1C1}} - \mu_{\text{A1B1C2}} - \mu_{\text{A1B2C1}} + \mu_{\text{A1B2C2}} - \\ && \mu_{\text{A2B1C1}} + \mu_{\text{A2B1C2}} + \mu_{\text{A2B2C1}} - \mu_{\text{A2B2C2}} \\ &= & 492 - 564 - 511 + 566 - 483 + 533 + 444 - 521 = -44 \end{array}$$

and we use the very same formula as before to calculate the variance for this three-way interaction:

24 $$\begin{array}{@{}rcl@{}} \sigma_{X_{\text{A:B:C}}}^{2} &=& 2^{k} \cdot \sigma^{2} - 2^{k} \cdot \sigma_{\text{cov}} \\ &=& 2^{3} \cdot 9000 - 2^{3} \cdot 7200 = 14400 \end{array}$$

#### Step 2: Performing the power analysis

Using these values, Cohen’s *d* for the three-way interaction is calculated as

25 $$\begin{array}{@{}rcl@{}} d_{X_{\text{A:B:C}}} &=& \frac{\mu_{X_{\text{A:B:C}}}}{\sigma_{X_{\text{A:B:C}}}} \\ &=& \frac{-44}{\sqrt{14400}} \approx -0.37 \end{array}$$

and we find that a sample size of *N* = 61 is needed to find an effect of this magnitude with a power of 1−β = .8.

### Excursus: The role of the correlation and the order of the interaction

#### Correlation among conditions

We can also express the variance of the difference variable in terms of the variances of the dependent variables and their correlation *ρ* (instead of their covariance):

26 $$\begin{array}{@{}rcl@{}} {\sigma_{X}^{2}} &= 2^{k} \cdot \sigma^{2} - 2^{k} \cdot \rho \cdot \sigma^{2} = 2^{k} \cdot \sigma^{2} (1 - \rho) \end{array}$$

This equation directly shows how the variance of the difference variable depends on the correlation between the dependent variables. In particular, the variance of the difference variable becomes smaller if the correlation is larger (i.e., 1 − *ρ* will decrease when *ρ* increases) and vice versa. This is especially important when performing a power analysis, because the effect size used for the power analysis depends on the variance of the difference variable. Looking back to the previous example in the Section “2 × 2 design: Interaction effect”, the variance of the difference variable was $\sigma _{X_{\text {A:B}}}^{2} = 7200$ and the corresponding effect size was $d_{X_{\text {A:B}}} \approx -0.68$. Recall that the correlation among the dependent variables is *ρ* = 0.8. However, if the correlation were only *ρ* = 0.2, the variance would be four times as large, that is, $\sigma _{X_{\text {A:B}}}^{2} = 2^{2} \cdot 9000 - 2^{2} \cdot 0.2 \cdot 9000 = 28800$, and thus the effect size would be only half the size $d_{X_{\text {A:B}}} = \frac {-58}{\sqrt {28800}} \approx -0.34$. The required sample size to achieve a power of 1−β = .8 for this case would dramatically increase from *N* = 19 to *N* = 70.

#### Order of effects

Another interesting fact when considering Eq. 26 is that the variance of the difference variable increases (and thus the effect size and power decrease) with the size of the design. The variance for the main and interaction effects in a 2 × 2 design is (with values taken from our example)

27 $$\begin{array}{@{}rcl@{}} \sigma_{X_{2: 2}}^{2} = 2^{2} \cdot 9000 - 2^{2} \cdot 0.8 \cdot 9000 = 7200 \end{array}$$

and is

28 $$\begin{array}{@{}rcl@{}} \sigma_{X_{2:2:2}}^{2} = 2^{3} \cdot 9000 - 2^{3} \cdot 0.8 \cdot 9000 = 14400 \end{array}$$

for a 2 × 2 × 2 design. The consequence is that the effect size decreases by the order of $\sqrt {2}$, which in turn has implications for statistical power and sample size calculations.

#### Implicit correlation between dependent variables

Finally, we would like to raise awareness about a possible mistake that researchers might commit. In particular, researchers might use the variance of the dependent variables’ variance *σ*^2^ when calculating the variance of the difference variable, thereby ignoring the correlation between the dependent variables. The reason for this could be that researchers do not have information about the covariance of the variables or they do not know how to perform the calculations properly. However, when calculating Cohen’s *d* of a difference variable, we must not assume that the variance of the difference variable is equal to the variance of the dependent variables. Instead, we have to calculate the variance based on the variances and covariances of the dependent variables. Failing to do so can have a dramatic impact on power calculations. This is because we implicitly make an assumption about the correlation when equating both variances:

29 $$\begin{array}{@{}rcl@{}} {\sigma_{X}^{2}} &=& 2^{k} \cdot \sigma^{2} (1 - \rho) \end{array}$$

30 $$\begin{array}{@{}rcl@{}} \text{set}\; {\sigma_{X}^{2}} = \sigma^{2} \Rightarrow \quad \sigma^{2} &=& 2^{k} \cdot \sigma^{2} (1 - \rho) \end{array}$$

31 $$\begin{array}{@{}rcl@{}} \Leftrightarrow~~ \quad \rho &=& \frac{2^{k} - 1}{2^{k}} \end{array}$$

That is, if we assume that ${\sigma _{X}^{2}}$ (the variance of the difference variable) and *σ*^2^ (the variance of the dependent variables) are equal when comparing two means, we implicitly assume a correlation of, e.g., *ρ* = 0.5 if *k* = 1. The correlation even increases for more complex designs. For difference variables of main and interaction effects in a 2 × 2 design, the correlation is *ρ* = 0.75, and for a 2 × 2 × 2 design, the correlation is *ρ* = 0.875. This might have a huge impact on sample size calculations, because – as we have seen earlier – the power is also a function of the correlation. As a consequence, the required sample size may be underestimated (or overestimated) if the true correlation is in fact lower (or higher) than the implied correlation.

For instance, consider the comparison of two means from the beginning of this section. The effect size was *d* = 0.15 and *N* = 351 were needed to detect this effect with a probability of 1−β = .8. Without taking into account the covariance between the RTs in the two conditions, we would assume the effect is $d = \frac {9}{\sqrt {9000}} \approx 0.09$, thus requiring a sample size of *N* = 875. The problem also occurs with higher-order interactions. In the 2 × 2 × 2 example right before this excursus, the effect size was *d* = − 0.37 and we would require *N* = 61 subjects to detect the effect with a probability of 1−β = .8. If we neglected the covariance, we would assume the effect is $d = \frac {-44}{\sqrt {9000}} \approx -0.46$, thus requiring a sample size of *N* = 39.

## Strategy 2 and 3: Effect size approach

The critical aspect when conducting power analyses via the *t* test is the correct specification of Cohen’s *d*. In the previous section, we have described a strategy that requires specifying the exact mean and covariance structure or knowing the correct *d* at a population level. In the present section, we will consider an “effect size approach”: A researcher might have an idea about the effect size of an interaction or a main effect (for an overview and a review of common effect size measures, see e.g., Bakeman, 2005; Carroll & Nordholm, 1975; Cohen, 1973; Keselman et al., 1998; Lakens, 2013; Levine & Hullett, 2002; Olejnik & Algina, 2000, 2003; Richardson, 2011; Steiger, 2004), and is now confronted with transforming these values to *d*.

Two cases can be distinguished. First, it could be that researchers have knowledge about an observed effect size (e.g., from a previous experiment or from a meta-analysis). In this case, the observed ${\widehat {\eta} _{p}^{2}}$ or ${\widehat {\omega} _{p}^{2}}$ value needs to be transformed into $\widehat d$. Second, one might formulate the expectations on ${\eta _{p}^{2}}$ at the population level (e.g., as a minimum effect size of interest), and then transform this value to *d*.^4^ Although both transformations are very similar, the calculations differ slightly. In the former, the transformation is done at the level of observed values, while in the latter the transformation is done at the level of population parameters. If sample sizes are large, both lead to approximately equal results though.

We begin by introducing the conversion on both levels for the simple case of comparing only two means of the whole design (similar to what we have done in Section “Comparing two means” of the previous section). Although it might not be very common to express an effect size in terms of ${\eta _{p}^{2}}$ in this case, this is of course possible and we can also use an *F*-test to compare the two means (i.e., a one-way ANOVA with one factor that has two levels). Importantly, the relation holds for main and interaction effects in multi-factorial repeated measures designs, which will be considered thereafter. The section will be finished by considering a tempting, but wrong, approach based on the semantic labels of effect sizes as suggested by Cohen (1988).

### Comparing two means

We first consider the sample level. In this case, the relation between ${\widehat {\eta} _{p}^{2}}$/${\widehat {\omega} _{p}^{2}}$ and Cohen’s $\widehat d$ for the within-subject case is (for more details on this, see Appendix A),

32 $$\begin{array}{@{}rcl@{}} \widehat d &= &\sqrt{\frac{{\widehat {\eta}_{p}^{2}} \cdot (N-1)}{N - {\widehat {\eta}_{p}^{2}}\cdot N}} \end{array}$$

33 $$\begin{array}{@{}rcl@{}} \widehat d &= &\sqrt{\frac{{\widehat {\omega}_{p}^{2}} N - {\widehat {\omega}_{p}^{2}} + 1}{N - {\widehat {\omega}_{p}^{2}}\cdot N} } \quad , \end{array}$$

where *N* indicates the sample size. For the example used in Section “Comparing two means” of the previous section, the effect size of that comparison can also be expressed as ${\widehat {\eta} _{p}^{2}} = 0.023$ or ${\widehat {\omega} _{p}^{2}} = 0$.^5^ Using Eq. 32, Cohen’s *d* is

34 $$\begin{array}{@{}rcl@{}} \widehat d = \sqrt{\frac{0.023 \cdot 35}{36 - 0.023\cdot 36}} = 0.15 \end{array}$$

matching exactly the previously calculated value. This conversion could be done with a simple R function as well: 
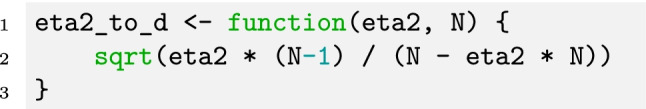


Calling this function as  yields $\widehat d = 0.15$.

Thus, having obtained some typical effect sizes in terms of ${\widehat {\eta} _{p}^{2}}$ from, for example, a literature review, we can transform those values to Cohen’s $\widehat d$, and use the result for sample size calculations and a power analysis using the *t* test. This can be done in the exact same way as described in Step 2 in the subsections of “Strategy 1: Means and covariances approach”: 
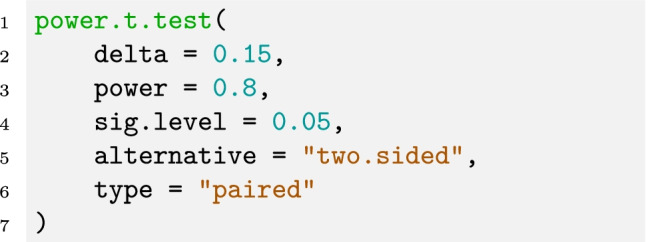


A single sample estimate for the effect size may be used when we only have limited knowledge about the true effect size, for instance, when there is just a single study at hand. Ideally, we should pool multiple estimates, of course. Anyhow, we should be aware that ${\widehat {\eta} _{p}^{2}}$, ${\widehat {\omega} _{p}^{2}}$, and Cohen’s $\widehat d$ overestimate the true effect size and we thus likely underestimate the sample size required to achieve a desired level of power (e.g., Mordkoff, 2019; Goulet-Pelletier & Cousineau, 2018).

In some instances, we might ”know” the effect size at the population level to perform a power analysis (e.g., by considering a minimum effect size of interest). Then, ${\eta _{p}^{2}}$ and ${\omega _{p}^{2}}$ are identical, and the transformation to Cohen’s *d* slightly changes to (for more details on this, see Appendix B):

35 $$\begin{array}{@{}rcl@{}} d = \sqrt{\frac{{\eta_{p}^{2}}}{1 - {\eta_{p}^{2}}}} \quad \end{array}$$

Note that this transformation is no longer dependent on the sample size, because it is based on the population effect size. If we “know” the true population effect size ${\eta _{p}^{2}}$, we should use Eq. 35 and perform the power analysis on its result. A simple R function to perform the transformation could be: 
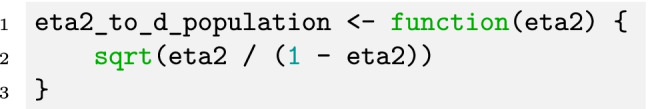


Calling the function with  yields *d* = 0.153. This value slightly differs from the previous transformation, because we assume that ${\eta _{p}^{2}} = .023$ matches the true effect size at the population level (i.e., we ignore the bias of the estimator).

### Main and interaction effects

The transformations shown in the previous section also hold true for main effects and interactions as long as the effect of interest can be expressed in terms of a *single* difference variable (i.e., the test has one numerator degree of freedom). In the previous section, we have already stated that all main and interaction effects in 2 × 2 ×... × 2 designs can indeed be expressed in terms of a single difference variable.

As an example, we use the three-way interaction based on Experiment 2 by Janczyk and Leuthold (2018), similar to what we have done in Section “2 × 2 × 2 design: Three-way interactions” of the previous section. For this effect, the effect size is ${\widehat {\eta} _{p}^{2}} = .118$. Applying Eq. 32, we obtain

36 $$\begin{array}{@{}rcl@{}} \widehat d = &\sqrt{\frac{0.118 \cdot 35}{36 - 0.118\cdot 36}} = 0.36 \end{array}$$

which again matches the value from Section “2 × 2 × 2 design: Three-way interactions” of the previous section. In the next step, we can then use this effect size and the *t* test to estimate the required sample size and perform a power analysis, just as it was done in the previous section.

Table 2 provides an excerpt of sample sizes required for achieving a power level of 1−β = .8 for different values of ${\eta _{p}^{2}}$ and ${\omega _{p}^{2}}$, respectively. It shall provide a quick way for researchers to conduct a priori power considerations for a main *or* interaction effect of interest.

**Table 2:** Required sample size to achieve a statistical power of 1−β = .8 given *α* = .05 and the effect size ${\eta _{p}^{2}}$ or ${\omega _{p}^{2}}$ in RM-ANOVA for effects with one numerator degree of freedom

	$${\eta _{p}^{2}}/{\omega _{p}^{2}}$$									
${\eta _{p}^{2}}/{\omega _{p}^{2}}$	.00	.01	.02	.03	.04	.05	.06	.07	.08	.09
0.0		779	387	256	191	152	125	107	93	82
0.1	73	66	60	55	51	47	44	41	38	36
0.2	34	32	30	29	27	26	25	24	23	22
0.3	21	20	19	18	18	17	16	16	15	15
0.4	14	14	13	13	13	12	12	11	11	11
0.5	10	10	10	10	9	9	9	9	8	8
0.6	8	8	7	7	7	7	7	7	6	6
0.7	6	6	6	6	5	5	5	5	5	5

Row names indicate the first decimal place of ${\eta _{p}^{2}}/{\omega _{p}^{2}}$, column names indicate the second decimal place. For instance, *N* = 26 subjects are required to achieve a power of 1−β = .8 for an effect size of ${\eta_{p}^{2}}/{\omega _{p}^{2}}= .25$ (third row, sixth column)

It is also noteworthy that this transformation holds for designs with factors with more than two levels – as long as the effect of interest consists of a subset of factors that only have two levels. Consider, for example, a 3 × 2 × 2 design (i.e., factor A has three levels, B has two levels, C has two levels). The main effects of factor B and C and the interaction effect B:C have only one numerator degree of freedom or, stated differently, the involved factors have only two levels each. For such effects, the relations outlined above hold true as well (for a tutorial on contrast coding in complex multi-factorial designs, see Schad, Vasishth, Hohenstein, & Kliegl, 2020).

### Converting effect sizes via Cohen’s semantic labels

Against the background from the previous sections, we finally consider a possible mistake that an incautious researcher may commit when converting ${\eta _{p}^{2}}$ to *d*: Wrongly applying the formulas for within-subject versus between-subject cases (see also Brysbaert, 2019).

Remember that, according to the suggestions of Cohen (1988), *d* = 0.2 is considered a small, *d* = 0.5 a medium, and *d* = 0.8 a large effect, while ${\eta _{p}^{2}} = .01$ is considered small, ${\eta _{p}^{2}} = .06$ medium, and ${\eta _{p}^{2}} = .14$ large. It is well known that small, medium, and large effect sizes indeed correspond to each other in the between-subject case with two groups, as this conversion was derived from Cohen’s *f* (see also Appendix C, and Cohen, 1988):

37 $$\begin{array}{@{}rcl@{}} d_{\text{between}} = 2f = 2\cdot\sqrt{\frac{{\eta_{p}^{2}}}{1-{\eta_{p}^{2}}}} \quad \end{array}$$

It is tempting to calculate the power for a “small”, “medium”, or “large” interaction in terms of ${\eta _{p}^{2}}$ by simply applying the semantic labels and choose the corresponding convention in terms of Cohen’s *d*. However, as should have become clear from this section (see in particular Eq. 35), a conversion according to Eq. 37 is not valid in the within-subject case, where the relation between *d* and ${\eta ^{2}_{p}}$ is instead (see Appendix D for the proof):

38 $$\begin{array}{@{}rcl@{}} d_{\text{within}} = f = \sqrt{\frac{{\eta_{p}^{2}}}{1-{\eta_{p}^{2}}}} \quad \end{array}$$

Consequently, a researcher would have to divide the expected *d* by two, and as a result, the required sample sizes often differ considerably. For instance, a researcher may expect a (large) effect size of ${\eta _{p}^{2}} = .14$ according to a recent meta-analysis in the field. Using the (wrongly assumed) corresponding large value of *d* = 0.8, results in a required sample size of *N* = 15 to achieve a power of at least 1−β = .8. The formula above, however, suggests to use *d* = 0.4 instead, which yields a sample size of *N* = 52.

## Discussion

Running well-powered experiments helps increasing the reproducibility of psychological research (see Ioannidis, 2005; Fraley & Vazire, 2014). Calculating the power, however, can be complicated as soon as one goes beyond the designs of simple *t* tests. Because of this, it is useful to conceive main and interaction effects of factors as “differences of differences”, which eventually can be treated in the framework of a *t* test.

In this tutorial article, we focused on exactly this situation and discussed how to use the *t* test to perform power analyses in multi-factorial repeated measures designs that involve factors with two levels only (i.e., hypothesis tests with one numerator degree of freedom). Two cases can be distinguished. In the first case, means, variances, and covariances of the dependent variable are available for each experimental condition and combinations thereof. In the second case, previously reported or expected effect sizes are available in terms of ${\eta ^{2}_{p}}$/${\widehat {\eta} ^{2}_{p}}$ or ${\widehat {\omega} ^{2}_{p}}$. In either case, a translation into $\widehat d$ or *d* is desired, and we demonstrated how to do so. To aid with the required calculations, we provide example R code in an online repository. Moreover, we developed an R package called *powerANOVA* (Langenberg, 2022) for this article that comes with a graphical user interface and implements the presented procedures.

### Key message

The good news is that functions calculating the power in the framework of *t* tests can indeed be used to calculate the power in 2 × 2 ×... within-subject designs. Yet, some measures of precaution are required to correctly perform the calculations.

Based on the means of the dependent variables, it is easy to establish the correct numerator of *d* when conceiving the effect of interest as a difference variable (e.g., as a “differences of differences” in case of interactions). The critical part is how to calculate the correct standard deviation for the denominator. Importantly, the correct value is not simply the (averaged) standard deviation within the conditions. Rather its correct calculation includes all variances within and the correlations/covariances between the conditions.

Nowadays, most scientific publications present means and some form of variability in figures and/or tables. However, converting standard errors back into variances is not straightforward when they are, for example, based on the error term from an ANOVA (Loftus & Masson, 1994). Furthermore, correlations/covariances of the dependent measures between the conditions are almost never reported. Thus, researchers likely have to set their (co-)variances/correlations based on a reasonable expectation or by calculating them from a raw data set. For this reason, we consider it helpful if researchers also report correlations/covariances (and perhaps even variances). With this information at hand, power analyses for interactions in 2 × 2 ×... × 2 designs can be performed straightforward.

Power analysis can also be performed based on typical effect sizes ${\widehat {\eta} ^{2}_{p}}$, ${\eta ^{2}_{p}}$, or ${\widehat {\omega} ^{2}_{p}}$ for the experimental context in which their study is embedded. These effect sizes may be derived from previous research, a meta-analysis, or simply be assumed. In this case, a relationship between those measures and Cohen’s $\widehat d$ or *d* exists. Yet, it would be wrong to simply equate them based on their semantic meaning as proposed by Cohen (1988) (i.e., as small, medium, or large effects). More precisely, if, for instance, a value ${\eta _{p}^{2}}=.14$ is assumed for the 2 × 2 interaction (thus a “large” effect), the correct value entered into functions calculating the power for *t* tests is not *d* = 0.8, but rather half of it (see Eq. 35).

### Limitations and further directions

The clearest limitation of this article is its scope on within-subject designs with factors of two levels each. Importantly, different factorial designs that include the same experimental manipulation may produce different effect size estimates if they include additional manipulations or covariates. For instance, imagine an experiment that investigates the CE in a Simon task and additionally includes the covariate *age*. In this case, the experiment may be able to explain a larger amount of variance as another experiment that uses the exact same design, but does not account for age. As a result, partial effect size estimates can differ. To resolve this issue, generalized effect size estimators have been developed, such as generalized *η*^2^ ($\eta ^{2}_{\text {G}}$). The present article does not cover generalized effect size estimators, but integrating our considerations in the context of generalized effect size estimators could be an interesting research question.

Power analysis is also a topic in multilevel models (MLM; also referred to as hierarchical models or random effects models; Fitzmaurice, Laird, & Ware, 2011; Laird & Ware, 1982), which have become increasingly popular in experimental cognitive psychology. Typically, replications in each condition are averaged within participants in order to be able to use RM-ANOVA (i.e., a single value per participant and condition). MLM models are able to include multiple replications per participant and to account for heterogeneity in main and interaction effects. This can ultimately increase statistical power as all available information can be used. There are a number of articles available that cover power analysis in multilevel models. For instance, Brysbaert and Stevens (2018) and Kumle, Võ, and Draschkow (2021) wrote two helpful tutorials (see also, Arend & Schäfer, 2019; DeBruine & Barr, 2021; Lafit et al., 2021). There are, furthermore, various software packages that can perform the necessary calculations (*mixedpower*, Kumle, Võ, & Draschkow, 2020; *simglm*, LeBeau, 2019; *pamm*, Martin, 2020; *simr*, Green & MacLeod, 2016; *powerlmm*, Magnusson, 2018). The packages *mixedpower* and *simr* are introduced in a comprehensive way by Kumle et al. (2021).

Replications across experimental conditions can also be incorporated using latent variable models. For instance, in a Simon task, replications in the congruent condition in trial *n* can be used as indicators to measure the RT in that condition more reliably. This way, measurement error can explicitly be modeled and main and interaction effects can then be tested on the latent variable level. Langenberg, Helm, and Mayer (2022) showed how to test main and interaction effects in RM-ANOVA using latent variables. It could also be an interesting research question how the calculations in this tutorial article generalize to latent variable models.

### Conclusions

In summary, this tutorial article aimed at deepening the understanding of main and interaction effects and how to express them in terms of difference variables, in an attempt to clarify whether or not the framework of a *t* test can be used to calculate power for interaction effects in within-subject designs. The required calculations to do so are covered in an extensive online repository.

## Appendix A: From ${\widehat {\eta} _{p}^{2}}$ and ${\widehat {\omega} _{p}^{2}}$ to Cohen’s $\widehat d$

Within RM-ANOVA, hypothesis tests are usually based on the sums of squares. The sums of squares are a measure for the part of the variance that can be attributed to the involved factors and interactions, and the part that can be attributed to error associated with the factors and interactions. The *F*-statistic is the ratio of mean sums of squares (Nesselroade & Cattell, 1988), that is,

39 $$\begin{array}{@{}rcl@{}} F = \frac{\textit{MS}_{\text{effect}}}{\textit{MS}_{\text{error}}} \quad , \end{array}$$

where *MS*_effect_ is the mean sum of squares from the main or interaction effect of interest (e.g., an interaction between factor A and factor B), and *MS*_error_ is the mean error sum of squares. Both are defined as the respective sums of squares divided by the corresponding degrees of freedom *d**f*_1_ (effect) and *d**f*_2_ (error):

40 $$\begin{array}{@{}rcl@{}} \textit{MS}_{\text{effect}} &=& \frac{\textit{SS}_{\text{effect}}}{df_{1}} \end{array}$$

41 $$\begin{array}{@{}rcl@{}} \textit{MS}_{\text{error}} &=& \frac{\textit{SS}_{\text{error}}}{df_{2}} \quad . \end{array}$$

The effect sizes ${\eta _{p}^{2}}$ and ${\omega _{p}^{2}}$ (e.g., Bakeman, 2005; Carroll & Nordholm, 1975; Cohen, 1973; Keselman et al., 1998; Lakens, 2013; Levine & Hullett, 2002; Olejnik & Algina, 2000, 2003; Richardson, 2011; Steiger, 2004) are estimated as:

42 $$\begin{array}{@{}rcl@{}} {\widehat {\eta}_{p}^{2}} & =& \frac{\textit{SS}_{\text{effect}}}{ \textit{SS}_{\text{effect}} + \textit{SS}_{\text{error}}} \end{array}$$

43 $$\begin{array}{@{}rcl@{}} {\widehat {\omega}_{p}^{2}} & = &\frac{df_{1} (\textit{MS}_{\text{effect}} - \textit{MS}_{\text{error}})} {\textit{SS}_{\text{effect}} + (N - df_{1}) \textit{MS}_{\text{error}}} \quad . \end{array}$$

The equations slightly differ as both estimators are biased to a different extent. ${\widehat {\omega} _{p}^{2}}$ is known to be less biased, ${\widehat {\eta} _{p}^{2}}$ is, however, used more often in practice (Okada, 2013; Richardson, 2011). For study designs that only involve factors with two levels each, the main and interaction effects have one numerator degree of freedom (i.e., *d**f*_1_ = 1). In this case, a simple relationship between *F* and *t* holds, as the empirical *F*-statistic is identical to the square of the empirical *t*-statistic:

44 $$\begin{array}{@{}rcl@{}} F = t^{2} \quad . \end{array}$$

Using these definitions and a relation between *t* and $\widehat d$, it is possible to translate the RM-ANOVA effect size measures into Cohen’s $\widehat d$. The reasoning is that, from Eqs. 39, 42, and 43, it can be seen that both the *F*-statistic as well as the effect size measures are a function of the sums of squares. By rearranging, the effect size measures can thus be expressed as a function of the *F*-statistic (Friedman, 1968; Kennedy, 1970), and with Eq. 44 they can be expressed as a function of the *t*-statistic (see also, Mordkoff, 2019):

45 $$\begin{array}{@{}rcl@{}} {\widehat {\eta}_{p}^{2}} &= & \frac{\textit{SS}_{\text{effect}}}{ \textit{SS}_{\text{effect}} + \textit{SS}_{\text{error}}}\\ &= & \frac{\textit{MS}_{\text{effect}}}{ \textit{MS}_{\text{effect}} + \textit{MS}_{\text{error}} \,\cdot\, df_{2}}\\ &= & \frac{F \cdot \textit{MS}_{\text{error}}}{F \,\cdot\, \textit{MS}_{\text{error}} + \textit{MS}_{\text{error}} \,\cdot\, df_{2}}\\ &= & \frac{F}{F + df_{2}}\\ &= & \frac{t^{2} }{t^{2} + df_{2}} \end{array}$$

46 $$\begin{array}{@{}rcl@{}} {\widehat {\omega}_{p}^{2}} &=& \frac{(\textit{MS}_{\text{effect}} - \textit{MS}_{\text{error}})}{\textit{SS}_{\text{effect}} + (N - 1) \textit{MS}_{\text{error}}}\\ &=& \frac{F \,\cdot\, \textit{MS}_{\text{error}} - \textit{MS}_{\text{error}}} {F \,\cdot\, \textit{MS}_{\text{error}} + (N - 1) \textit{MS}_{\text{error}}}\\ &=& \frac{F - 1} {F + N - 1}\\ &=& \frac{t^{2} - 1}{t^{2} + N - 1} \quad . \end{array}$$

In case *d**f*_1_ = 1, *d**f*_2_ equals *N* − 1 and thus the denominators of Eqs. 45 and 46 are equivalent. However, the numerators slightly differ. We can now use the relation

$$\begin{array}{@{}rcl@{}} t = \widehat d \sqrt{N} \end{array}$$

and plug it into Eqs. 45 and 46. Solving for $\widehat d$ yields:

47 $$\begin{array}{@{}rcl@{}} \widehat d &= &\sqrt{\frac{{\widehat {\eta}_{p}^{2}} \cdot {(N-1)}}{N - {\widehat {\eta}_{p}^{2}}\cdot N}} \end{array}$$

48 $$\begin{array}{@{}rcl@{}} \widehat d &= &\sqrt{\frac{{\widehat {\omega}_{p}^{2}} N - {\widehat {\omega}_{p}^{2}} + 1}{N - {\widehat {\omega}_{p}^{2}}\cdot N} } \quad . \end{array}$$

Although the equations differ, the calculations will lead to the same Cohen’s $\widehat d$.

## Appendix B: From ${\eta _{p}^{2}}$ and ${\omega _{p}^{2}}$ to *d* at the population level

When effect sizes need to be formulated directly at the population level, for instance, by defining a minimum ${\eta _{p}^{2}}$ that is considered relevant, ${\eta _{p}^{2}}$ has to be transformed into *d*. To this end, ${\eta _{p}^{2}}$ and ${\omega _{p}^{2}}$ is defined at the population level for the example with one numerator degree of freedom as:

49 $$\begin{array}{@{}rcl@{}} {\eta_{p}^{2}} = {\omega_{p}^{2}} = \frac{{\mu^{2}_{X}} }{{\mu^{2}_{X}} + {\sigma^{2}_{X}}} \quad , \end{array}$$

where *μ*_*X*_ and *σ*_*X*_ are the expected value and standard deviation of the interaction variable when it is expressed as a “difference of differences” (see Section “Strategy 1: Means and covariances approach”, for more details, see also Appendix D). Note that ${\eta _{p}^{2}}$ is equal to ${\omega _{p}^{2}}$ as both effect size measures are the same at the population level, only their estimators from the sample are different. By extending Eq. 49 by ${\sigma _{X}^{2}}$, one can rearrange it as a function of *d*:

$$\begin{array}{@{}rcl@{}} {\eta_{p}^{2}} &= & \frac{{\mu^{2}_{X}}}{{\mu^{2}_{X}} + {\sigma^{2}_{X}}} \cdot \frac{{\sigma_{X}^{2}}}{{\sigma_{X}^{2}}} \\ &= & \frac{\frac{{\mu^{2}_{X}}}{{\sigma^{2}_{X}}}}{\frac{{\mu^{2}_{X}}}{{\sigma^{2}_{X}}} +\frac{{\sigma^{2}_{X}}}{{\sigma^{2}_{X}}}} \\ &= & \frac{d^{2}}{d^{2} + 1} \\ \end{array}$$

Solving for *d* then yields the conversion from ${\eta _{p}^{2}}$ to *d* (see also Brysbaert, 2019):

50 $$\begin{array}{@{}rcl@{}} d_{\text{within}} = \sqrt{\frac{{\eta_{p}^{2}}}{1 - {\eta_{p}^{2}}}} \quad . \end{array}$$

Two things are worth pointing out. First, the transformations expressed at the sample and population level are related to each other. Except for *d**f*_2_ and *N*, Eq. 50 at the population level is identical to Eq. 47 at the sample level. In fact, if we consider that *d**f*_2_ gets larger with *N*, both are approximately equal. Second, the derived equations differ from the common conversion of ${\eta _{p}^{2}}$ into Cohen’s *d* for between-subject designs (see Appendices C and D). For between-subject designs ${\eta _{p}^{2}}$ translates to *d* as

51 $$\begin{array}{@{}rcl@{}} d_{\text{between}} = 2 \cdot \sqrt{\frac{{\eta_{p}^{2}}}{1 - {\eta_{p}^{2}}}} \quad . \end{array}$$

Thus, the same effect size in terms of ${\eta _{p}^{2}}$ translates differently to Cohen’s *d* for within- and between-subject designs. However, *d* for the within-subject case is only half as large as *d* for the between-subject case, which can dramatically change power calculations (see also Brysbaert, 2019, for the same argument).

## Appendix C: Cohen’s ***d*** and ***f*** in between-subject designs

This appendix details the relationship between Cohen’s *f*^2^, ${\eta _{p}^{2}}$, and *d* for the between-subject case at the population level (Cohen, 1988). To this end, let us first state the formula for *f*^2^ and ${\eta _{p}^{2}}$:

52 $$\begin{array}{@{}rcl@{}} f^{2} &=& \frac{\sigma_{\text{effect}}^{2}}{\sigma^{2}} \end{array}$$

53 $$\begin{array}{@{}rcl@{}} {\eta_{p}^{2}} &=& \frac{\sigma_{\text{effect}}^{2}}{\sigma_{\text{effect}}^{2} + \sigma^{2}} \quad , \end{array}$$

where $\sigma _{\text {effect}}^{2}$ is the variance attributable to the effect of interest, and *σ*^2^ is the error variance. The general relationship between *f*^2^ and ${\eta _{p}^{2}}$ is straightforward and well known. Rewriting Eq. 52 in terms of $\sigma _{\text {effect}}^{2}$ and plugging this into Eq. 53 yields

$$\begin{array}{@{}rcl@{}} {\eta_{p}^{2}} = \frac{f^{2} \sigma^{2}}{f^{2} \sigma^{2}+ \sigma^{2}} = \frac{f^{2}}{f^{2} + 1} \quad . \end{array}$$

Solving this equation for *f*^2^ then yields

$$\begin{array}{@{}rcl@{}} f^{2} = \frac{{\eta_{p}^{2}}}{1 - {\eta_{p}^{2}}} \quad . \end{array}$$

Now consider the relationship between *f*^2^ and *d* in a simple two-group between-subject design (i.e., the standard two-sample *t* test). Here, $\sigma _{\text {effect}}^{2}$ is the sum of squared differences between the population means *μ*_*i*_ (*i* ∈{1, 2}) and the grand mean *μ* (i.e., the average deviation from the grand mean)

$$\begin{array}{@{}rcl@{}} \sigma_{\text{effect}}^{2} = \frac{1}{2}\sum\limits_{i=1}^{2}(\mu_{i} - \mu)^{2} = \frac{1}{2}\sum\limits_{i=1}^{2}\left(\mu_{i} - \frac{\mu_{1} + \mu_{2}}{2}\right)^{2} \quad , \end{array}$$

and *σ*^2^ is the variance within each group. We can therefore express *f*^2^ as:

$$\begin{array}{@{}rcl@{}} f^{2} = \frac{\frac{1}{2}{\sum}_{i=1}^{2}\left(\mu_{i} - \frac{\mu_{1} + \mu_{2}}{2}\right)^{2}}{\sigma^{2}} \quad . \end{array}$$

The key is now to rewrite this equation in a way that it involves $d = \frac {\mu _{1} - \mu _{2}}{\sigma}$:

$$\begin{array}{@{}rcl@{}} \Rightarrow f^{2} &=& \frac{\frac{1}{2} \left[ \left(\mu_{1} - \frac{\mu_{1}+\mu_{2}}{2}\right)^{2} + \left(\mu_{2} - \frac{\mu_{1}+\mu_{2}}{2}\right)^{2} \right]}{\sigma^{2}} \\ &=& \frac{\frac{1}{2} \left[ \left(\frac{2\mu_{1} - \mu_{1}-\mu_{2}}{2}\right)^{2} + \left(\frac{2\mu_{2} - \mu_{1} - \mu_{2}}{2}\right)^{2} \right]}{\sigma^{2}} \\ &=& \frac{\frac{1}{2} \left[ \left(\frac{\mu_{1} - \mu_{2}}{2}\right)^{2} + \left(\frac{\mu_{2} - \mu_{1}}{2}\right)^{2} \right]}{\sigma^{2}} \\ &=& \frac{\frac{1}{2} \left[ \left(\frac{\mu_{1} - \mu_{2}}{2}\right)^{2} + \left(\frac{\mu_{2} - \mu_{1}}{2}\right)^{2} \right]}{\sigma^{2}} \\ &=& \frac{1}{4} \frac{(\mu_{1} - \mu_{2})^{2}}{\sigma^{2}} = \frac{1}{4} d^{2} \\ \Leftrightarrow f & =& \frac{1}{2} d\quad \text{or} \quad d = 2f \end{array}$$

The original effect size categories suggested by Cohen (1988) state that a small effect accounts for 1% of the variance, a medium effect accounts for 6%, and a large effect accounts for 14% in terms of ${\eta _{p}^{2}}$. The categories for Cohen’s *d* were then derived using the above formula, which gives $d = 2\sqrt {\frac {0.01}{1-0.01}} \approx 0.2$, $d = 2\sqrt {\frac {0.06}{1-0.06}} \approx 0.5$, and $d= 2\sqrt {\frac {0.14}{1-0.14}} \approx 0.8$.

## Appendix D: Cohen’s ***d*** and ***f*** in within-subject designs

This appendix shows the relation between Cohen’s *f* and *d* for within-subject designs. Again, Cohen’s *f* and ${\eta ^{2}_{p}}$ are defined as:

54 $$\begin{array}{@{}rcl@{}} f^{2} &=& \frac{\sigma_{\text{effect}}^{2}}{\sigma^{2}} \end{array}$$

55 $$\begin{array}{@{}rcl@{}} {\eta_{p}^{2}} &=& \frac{\sigma_{\text{effect}}^{2}}{\sigma_{\text{effect}}^{2} + \sigma^{2}} \quad , \end{array}$$

where $\sigma ^{2}_{\text {effect}}$ is the variance attributable to the effect of interest, and *σ*^2^ is the error variance. For within-subject designs, the relation between *f* and ${\eta ^{2}_{p}}$ is the same as for between-subject designs, that is, $f^{2} = \frac {{\eta _{p}^{2}}}{1 - {\eta _{p}^{2}}}$. However, the relation between *f* and Cohen’s *d* is different. $\sigma ^{2}_{\text {effect}}$ is the squared mean of the difference variable and *σ*^2^ is the variance of the difference variable. For instance, for a one-way design with two levels, $\sigma ^{2}_{\text {effect}}$ is given by:

$$\begin{array}{@{}rcl@{}} \sigma^{2}_{\text{effect}} = {\mu_{X}^{2}} = (\mu_{Y_{\text{A1}}} - \mu_{Y_{\text{A2}}})^{2} \quad , \end{array}$$

and *σ*^2^ is given by:

$$\begin{array}{@{}rcl@{}} \sigma^{2} = {\sigma^{2}_{X}} = {\sigma_{1}^{2}} + {\sigma_{2}^{2}} - 2 \sigma_{1,2} \quad , \end{array}$$

where ${\sigma _{1}^{2}}$ and ${\sigma _{2}^{2}}$ are the variances within each condition, and *σ*_1,2_ is the covariance between conditions. Consequently, *f* can be expressed in terms of *d*:

$$\begin{array}{@{}rcl@{}} \Rightarrow f^{2} &=& \frac{\sigma_{\text{effect}}^{2}}{\sigma^{2}} \\ &=& \frac{{\mu^{2}_{X}}}{{\sigma^{2}_{X}}} = d^{2} \\ \Leftrightarrow f &=& d \quad \end{array}$$

It follows that, in the within-subject case, a small effect accounting for 1% of the variance yields $d = \sqrt {\frac {0.01}{1-0.01}} \approx 0.1$, a medium effect gives $d = \sqrt {\frac {0.06}{1-0.06}} \approx 0.25$, and a large effect gives $d = \sqrt {\frac {0.14}{1-0.14}} \approx 0.4$.

## Footnotes

1 Schäfer and Schwarz (2019) analyzed studies with and without pre-registration and compared their sample sizes. For between-subject designs, sample sizes were larger for studies with pre-registration. In contrast, for studies using within-subject designs, sample sizes were smaller for studies with pre-registration. The authors attributed the former result to a more sensible use of power analyses. For the latter (unexpected) result they tentatively suggested that power analyses revealed a smaller sample size.

2 In the following, “hats” will indicate estimators from a sample and symbols without a “hat” will indicate population parameters.

3 ${\widehat {\eta} _{p}^{2}}$ and ${\widehat {\omega} _{p}^{2}}$ are not to be confused with (generalized) ${\widehat {\eta} _{G}^{2}}$ and ${\widehat {\omega} _{G}^{2}}$. The latter two measures have been proposed to make effect sizes comparable across studies with different designs (e.g., Fleiss, 1969; Olejnik & Algina, 2003).

4 Note that ${\eta _{p}^{2}}$ is equal to ${\omega _{p}^{2}}$ at the population level

5 Note that, if the effect of interest is small, ${\widehat {\omega} _{p}^{2}}$ can turn out negative. In this case, ${\widehat {\omega} _{p}^{2}}$ is set to zero. This is the reason why the result of $\widehat d = 0.167$, which we obtain using Eq. 33 does not match with $\widehat d = 0.15$ from Eq. 32. If ${\widehat {\omega} _{p}^{2}}$ equals zero, then $\widehat d$ equals $\frac {1}{\sqrt {N}}$, and thus $\frac {1}{\sqrt {N}}$ is a lower bound for $\widehat d$ when converting from ${\widehat {\omega} _{p}^{2}}$. Conversely, when converting from $\widehat d$ to ${\widehat {\omega} _{p}^{2}}$, ${\widehat {\omega} _{p}^{2}}$ will be negative if $\widehat d < \frac {1}{\sqrt {N}}$.
